# Study of the Wound-Healing Activity of a New Drug Derived from Cobalt Polyacrylate

**DOI:** 10.3390/ijms26030899

**Published:** 2025-01-22

**Authors:** Anna P. Vasilyeva, Andrey V. Svinarev, Vladimir A. Ogurtsov, Evgeny N. Khodot, Oleg A. Rakitin, Elena V. Trubnikova, Elena S. Shcherbakova, Maria S. Smirnova, Victoria V. Shishkina, Tatyana V. Samoylenko, Alexei B. Shevelev

**Affiliations:** 1 Vavilov Institute of General Genetics RAS, Gubkin Str. 3, GSP-1, 119991 Moscow, Russia; bidja5hatma@gmail.com (A.P.V.); vao@ioc.ac.ru (V.A.O.); khodot@ioc.ac.ru (E.N.K.); ledera@yandex.ru (E.S.S.); mbarbotko@ya.ru (M.S.S.); shevel_a@hotmail.com (A.B.S.); 2 Innotech-21 LLC, Lyubertsy, Prospect Oktyabrsky, 145, VI, 140000 Moscow, Russia; penostroy@mail.ru; 3 Zelinsky Institute of Organic Chemistry of the Russian Academy of Sciences, Leninsky Prospect, 47, 119334 Moscow, Russia; orakitin@ioc.ac.ru; 4 Research Institute of Experimental Biology and Medicine, Department of Histology, Burdenko Voronezh State Medical University, Studencheskaya Str. 10, 394036 Voronezh, Russia; 4128069@gmail.com (V.V.S.); antailkka@mail.ru (T.V.S.)

**Keywords:** polyacrylate, cobalt, resveratrol, dexpanthenol, wound healing, Hestatin, mast cells

## Abstract

Previously we suggested a new pharmaceutical derived from coordination complex of Co^3+^ with polyacrylic acid (PAA) exhibiting hemostatic and microbicidal activity, namely Hestatin. Differences in the physiological activity of Hestatin synthesized from PAA 10 kDa (Hestatin 10) and 200 kDa (Hestatin 200) were shown. We tested the acute toxicity of Hestatin and its effect on the healing rate of sterile wounds in rats. Free 10 kDa PAA, emulsion wax, emulsion wax carrying resveratrol, and dexpanthenol were tested for comparison. Hestatin 10 exhibited no acute toxicity when administered intragastrically at dosages of 5 g per kg. Hestatin 10 surpassed all tested drugs in its wound healing ability. Histological analysis of skin sections of rats in the area of healing defects showed an increased rate of synthesis of reticular fibers compared to the placebo. In the early stages of wound healing (inflammatory phase), Hestatin 10 stimulated taxis of mast cells (MCs) to the wound bottom but not to the wound perimeter. At the final stage of wound healing (remodeling phase), Hestatin 10 promoted MC evacuation from the skin defect area. This effect is the opposite of the well-known wound-healing agents (dexpanthenol and resveratrol), which enhance MC infiltration into the defect area in the remodeling phase.

## 1. Introduction

Recently, we reported a novel pharmaceutical substance Hestatin derived from a tight coordination complex of Co^3+^ with polyacrylic acid (PAA) which exhibited a pronounced blood-clotting activity on the model of capillary and parenchymatous bleeding [1]. In the course of testing the hemostatic properties of Hestatin, we noted that beyond its principal activity, it promoted the healing of the wounds and prevented suppuration. Therefore, in this study, we aimed to confirm and quantitatively characterize the wound-healing efficacy of Hestatin and to describe the mechanism of its action at the cellular level.

A previous study demonstrated that the hemostatic properties of Hestatin strongly depended on the molecular weight (MW) of the polyacrylic acid (PAA) used for its synthesis [1]. Hestatin 10 (MW_PAA_ = 10 kDa) and Hestatin 200 (MW_PAA_ = 200 kDa), the tested variants with the minimal and the maximal MW, showed the highest hemostatic activity, whereas PAA samples with intermediate MWs of 50 kDa and 100 kDa were inefficient. Furthermore, experiments conducted on rabbits indicated that wounds treated with Hestatin healed faster and resulted in smaller scars compared to untreated wounds. In the present study, we investigated the mechanism behind the wound-healing effects of Hestatin 10 and Hestatin 200. Bepanthen ointment derived from dexpanthenol [2] in combination with Levomecol ointment (contains antibiotic chloramphenicol) was used as an acknowledged wound-healing drug. Resveratrol synthesized by a novel method with a reduced organophosphorus contamination was chosen as another reference. Resveratrol is widely known for its wound-healing activity [3] as well as its ability to stimulate post-ischemic myocardial recovery and to relieve the symptoms of metabolic syndrome [4]. It was also reported to exhibit antitumor activity [5,6] and to suppress the growth of pathogenic bacteria in vitro [7] and in vivo [8]. Taking into account the moderate hydrophobicity of the resveratrol contrasting to the entirely hydrophilic properties of Hestatin, we hypothesized that these two substances may complement each other within a complex wound-healing agent and where each of them may alternately stimulate one of the key stages of the wound-healing process. Therefore, we compared the wound-healing effect of Hestatin, resveratrol, Bepanthen, and other agents at the cellular level. Because the resveratrol is poorly soluble in water, a preparation containing 1% resveratrol delivered using emulsion wax was tested, along with a pure emulsion wax (used as another variant of the placebo).

The average daily reduction rate of the wound area and histological examinations of the wounds on the 5th, 10th, and 14th days after the inflicting the wounds were assessed under the study. Statistical analysis was used for estimating the confidence of the differences between the groups treated with different agents including the placebo. Taking into account the non-linear manner of the wound defect reduction and experimental errors of the wound square determination caused with a bias between the real wound defect and the fibrin clot square, we measured the effectiveness of the treatments by calculating the average daily reduction rate of wound area until the wound reached one of three alternative thresholds: undetectable size, less than 1 mm^2^, or less than 2 mm^2^. Contouring of the wound edge was carried out by three independent operators. The wound healing rates measured by all these methods were averaged.

Furthermore, we carried out histological examinations of the wounds on the 5th, 10th, and 14th days after the experiment began to assess various aspects, such as inflammatory infiltration, neovascularization, the presence of fibroblastic cells and macrophages, the presence and degranulation of MCs in the bottom and perimeter of the wounds, and the presence of reticular and collagen fibers.

Taking into account the uneven rate of the sterile wound healing at different stages (the initial inflammation period with a zero rate of decrease in the wound area followed by a fast wound-contracting stage and, finally, a slow stage of eventual defect elimination), standard statistic methods often fail to confirm the efficacy of the acknowledged wound-healing agents. Moreover, accurately measuring the wound area in an experiment is often impossible since the non-transparent blood clot protrudes beyond the edges of the wound, precluding the contouring of the defect. From a formal point of view, the fibrin clot falling off at the end of the contraction phase looks like simultaneous wound healing, although in fact the processes of restoring normal tissue under the clot in the defect lasts for much longer.

## 2. Results

### 2.1. Preparation of Samples for Testing

In the literature, an effective four-stage method for the chemical synthesis of resveratrol has been described. This method allows for a base substance with a purity of more than 96% without purification to be obtained [9]. By using trimethylphosphite instead of triethylphosphite in the second stage of the resveratrol synthesis scheme (Formula (1)), it was found that the reaction time and temperature were significantly reduced (from 4 h at 160 °C to 15 min at 120 °C) while the yield increased from 90 to 94%. Although the yield of all stages was close to the quantitative yield, resulting in a sample with the expected purity, analysis revealed that it contained about 1% impurities of organophosphorus compounds. These impurities may have toxicity when using the sample in biological experiments and cannot be disposed of by crystallization of the product from isopropyl alcohol according to the previously described procedure [10]. Efforts have been made to reduce the content of potentially dangerous impurities in the resveratrol sample by converting it to a tris-acetyl derivative followed by its hydrolysis. The tri-acetyl derivative of resveratrol (1-(3,5-diacetoxystyrol)-4-acetoxybenzene) was obtained by acylating the resveratrol with acetic anhydride in the presence of triethylamine in methylene chloride with a quantitative yield, as described in the detailed procedure for performing this stage of synthesis [11]. The resulting compound was subjected to hydrolysis without additional purification.


(1)

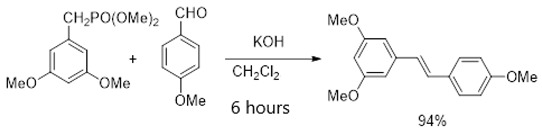



The tris-acetyl derivative of resveratrol was hydrolyzed using small amounts of sodium methylate in a mixture of tetrahydrofuran and methanol at room temperature for 2 h, rendering a 99% yield (Formula (2)). The procedure for this synthesis stage was previously described [12]. The resulting compound was then purified by crystallizing the product from isopropyl alcohol, following the method described previously [10].


(2)

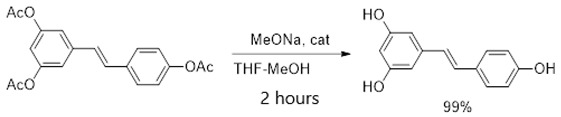



The structure of the synthesized substance was confirmed using elemental analysis, IR, NMR 13C, 1H spectroscopy, and chromatography–mass spectrometry. These data were compared with those reported previously. Our NMR 31P spectroscopy data indicated the absence of impurities of any organophosphorus compounds in the sample. The purity of the product, as determined by chromatography–mass spectrometry and 1H NMR spectroscopy, exceeded 99.8%.

Despite the abundance of data on resveratrol synthesis methods, we successfully modified the synthesis method by using trimethylphosphite instead of triethylphosphite in the second stage. This modification significantly reduced the reaction time and increased the yield. We also developed a novel method for the final purification of resveratrol from traces of organophosphorus compounds that could interfere with biological tests. This involved converting it to a tris-acetyl derivative followed by the hydrolysis of the resulting product. Furthermore, the yield of resveratrol in this purification method remained nearly quantitative (99%).

As a result of these efforts, we accumulated over 200 g of resveratrol of the required purity, of which more than 25 g were used for biological activity testing.

Cobalt polyacrylate samples were created from PAA with Mr = 9356 and 184,631 Da, as previously described [1]. PAA with Mr = 9356 was used as a comparison sample. These samples were used in animal testing as 10 g/L solutions in deionized water.

The synthesis of resveratrol (3,5,4′-trihydroxy-trans-stilbene) for testing was performed as per the procedure outlined in the Section 4. The total yield of the target substance using the proposed method was 53%. The synthesized resveratrol was dissolved in DMSO at a concentration of 250 g/L, and the resulting concentrated solution was then utilized to create a working solution based on emulsion wax, as detailed in the Materials and Methods section. A pure emulsion wax containing no active substance was used for comparison.

A commercial wound-healing agent Bepanthen ointment for external use by GP Grenzach Productions GmbH (Grenzach-Wyhlen, Germany) in combination with Levomecol was used as a positive control.

### 2.2. Testing Acute Toxicity of the Experimental Samples

The trials revealed that when Hestatin 10 was administered intragastrically to mice, and when Bepanthen by GP Grenzach Productions GmbH (Germany) was also administered intragastrically at a dose of 5 g/kg, no toxic effects or deaths of mice were observed. This information is summarized in Table 1.

The trials showed that administration of Hestatin 10 to mice through the stomach did not cause notable changes in their behavior, even when using the maximum dose (5 g/kg) limited by the subtance solubility and the stomach capacity. It was impossible to calculate the LD_50_ of the tested preps at this maximum volume allowed for intragastric administration to this type of animal. The trials also indicated that intragastric administration of Bepanthen by GP Grenzach Productions GmbH (Germany) to the mice did not cause significant signs of intoxication at the same dose of 5 g/kg.

The experimental data obtained by intragastric administration of Hestatin 10 to both male and female mice indicate that this drug can be classified as Class IV (low-toxicity medicinal substances). The condition of the animals that survived high-dosage administration of the tested substances indicated the good tolerability and harmlessness of Hestatin 10 in doses exceeding the therapeutic amounts by one hundred times. Hestatin 10, when studying its acute toxicity with intragastric administration to both male and female mice, showed the same toxicity as Bepanthen by GP Grenzach Productions GmbH (Germany).

### 2.3. Studies of the Wound-Healing Activity of Drugs on a Model of Sterile Wounds in Rats

Aqueous solutions of the tested preparations (O1, O2, O3, and O7) were applied to a wound using an automated pipet in an amount of 0.2 mL. Cotton swabs or other absorbent materials were not used. After applying the solutions, the animal was held motionless for 60 s before being turned back into an individual cage, with care taken to prevent the preparation from rolling off the wound and down the rat’s back. The preparations in the ointment form (O4, O5, and O6) were applied using a sterile cotton swab directly to the wound and to the surrounding skin. Laboratory animals were processed and photographed within a strictly controlled time according to their serial number. This was required to ensure the same intervals between applications of the medicines. On the first day of the experiment, the following manipulations were carried out: (1) applying model wounds to an anesthetized rat; (2) taking photos of the model wound with a ruler in the frame and determining the wound area with ImitoMeasure software (V.2.0.0.18, imito AG (initial value of the wound defect); (3) treatment of the wound surface with the tested medicines. All subsequent days: (1) taking photos of the model wound along with a ruler added to a square and measurement of the residual wound defect square with ImitoMeasure software; (2) treatment of the wound surface with the tested medicine.

To evaluate the effect of the tested preparation on the rate of wound healing, the decrease in the wound area in mm^2^ per day was used as a criterion. This was determined over time, starting from the second day of the experiment, when the fibrin clot on the wound was fully formed, until the average diameter of the remaining defect area did not exceed any of the following three thresholds: undetectable size, <1 mm^2^, or <2 mm^2^. It was noted that determining the time required to completely close the defect was technically difficult and poorly reproducible. This was because, with a defect area of less than 1 mm^2^, different observers assessed the presence or absence of a defect in the same animal at the same time point in different ways. Three operators were involved in the study for contouring the initial wound square in order to prevent subjective bias. Information about the samples sent for the testing of wound-healing activity is shown in Table 2.

**Figure 1:**
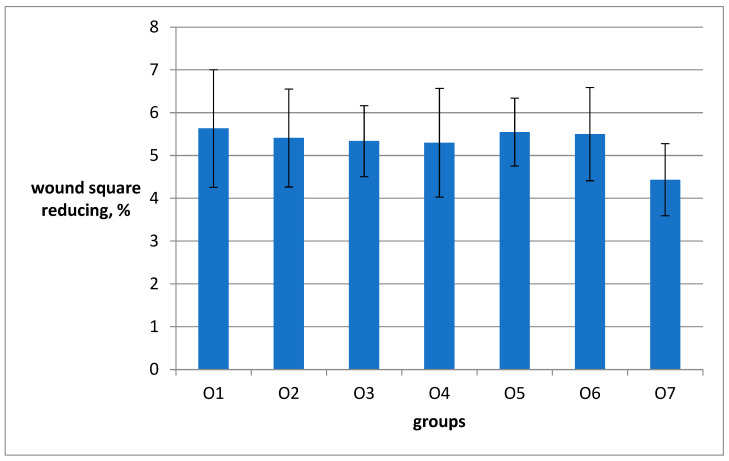
Rates of the decrease in the area of wounds in the animal groups. The vertical bars above the columns show the standard error for each group.

During the observation of wound healing, it was noticed that the peak of the inflammatory reaction occurred on the third day after the wound was inflicted. On the 5th and 10th days, the tissue remodeling phase was observed, and on the 14th day, the restoration of hair follicles was observed. Among the animal groups, O6 (emulsion wax), O5 (resveratrol), and O6 (Bepanthen) showed significantly worse external wound conditions compared to the groups O1, O2, and O3, where aqueous solutions were used. After the application of ointment and emulsion, the rats exhibited more restlessness and engaged in active grooming to clean the wounds and remove the applied substances from their fur. In the O5 group (resveratrol), the scabs were the most combed and visually larger, resulting in slower healing. By the 11th day of the experiment, there were no scabs on the wounds in groups O1, O2, O3, and O7, and the scars from the wounds were barely noticeable. In the examination of sections stained with hematoxylin and eosin, a significantly higher density of hair follicles was observed in animals of groups O1 (Hestatin 10) and O2 (Hestatin 200) compared to the placebo group (O7). Additionally, photographs of wounds at various periods of the experiment and primary measurement data of the residual area of the defect are provided in the Additional Materials. The results of the wound healing rate calculations are presented in Table 3 and Figure 1.

**Figure 2:**
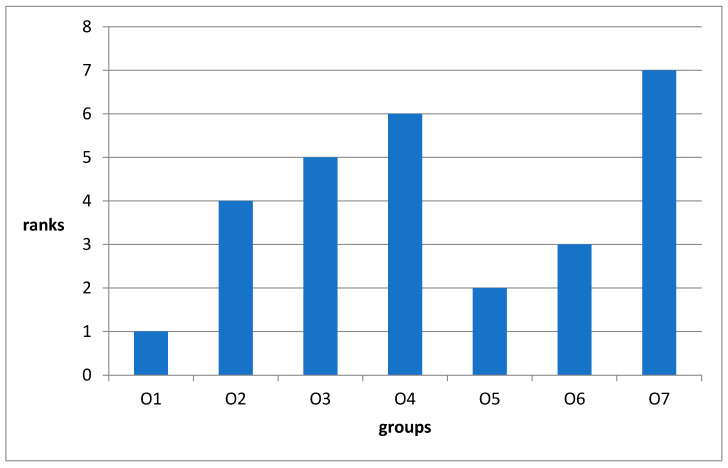
Rankings of the effectiveness of the tested drugs on the rate of wound healing.

Figure 2 shows the ranks of the effectiveness of the effect of the tested drugs on the rate of wound healing.

The reliability of the data obtained during the tests is confirmed by the fact that the control group O7 (placebo) had the lowest rate of wound healing among all groups. The rankings of the effectiveness of the tested drugs on the rate of wound healing (Figure 2) allowed us to conclude that the preparations O1 (Hestatin 10) and O5 (resveratrol) were more effective than the positive control O6 (Bepanthen). However, one should keep in mind that the wound-healing effect in O6 in contrast to O1 and O5 preparations was complemented by disinfection of the wound with the antibiotic ointment Levomecol.

It is noteworthy that the O2 (Hestatin 200) sample was less effective than the O6 comparison drug. An unexpected result of the tests is the pronounced wound-healing activity of the sample O3 (PAA) used to produce the Hestatin. This accelerated wound healing significantly compared to the placebo (O7). The worst indicator of the effect on the rate of wound healing was shown by sample O4, containing the free emulsion wax, which is fully in line with our expectations. In general, the experiment allows the conclusion that Hestatin 10 is highly effective as a wound-healing agent.

Along with the resveratrol delivered using emulsion wax, it surpassed the world’s best analog, Bepanthen ointment derived from dexpanthenol in combination with the antimicrobial medicine Levomecol, recommended for accelerating wound healing of various origins. It should be noted that in addition to accelerating wound healing, the new formula has shown the ability to reduce the level of wound inflammation and pain syndrome, which has serious independent significance for patients.

### 2.4. Study of the Mechanism of Action of Hestatin by Histochemical Analysis of Sections of the Healing Wounds

**Figure 3:**
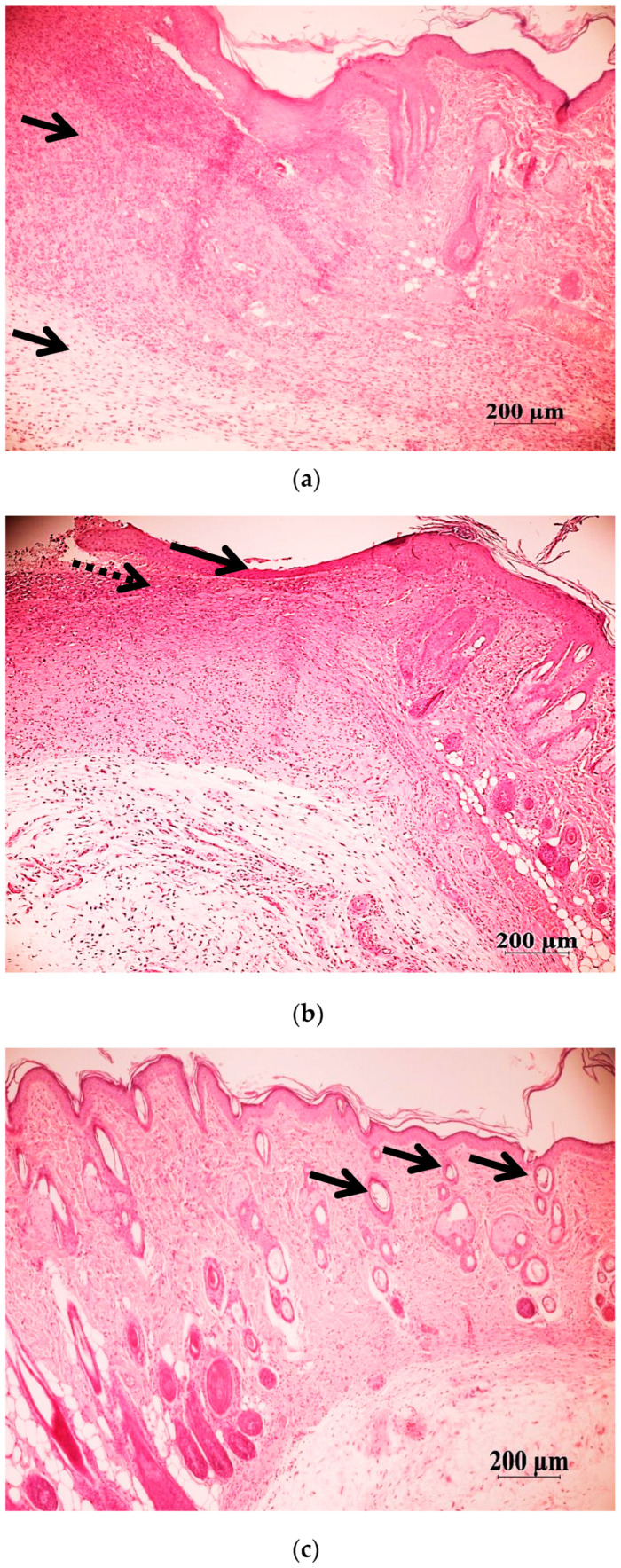

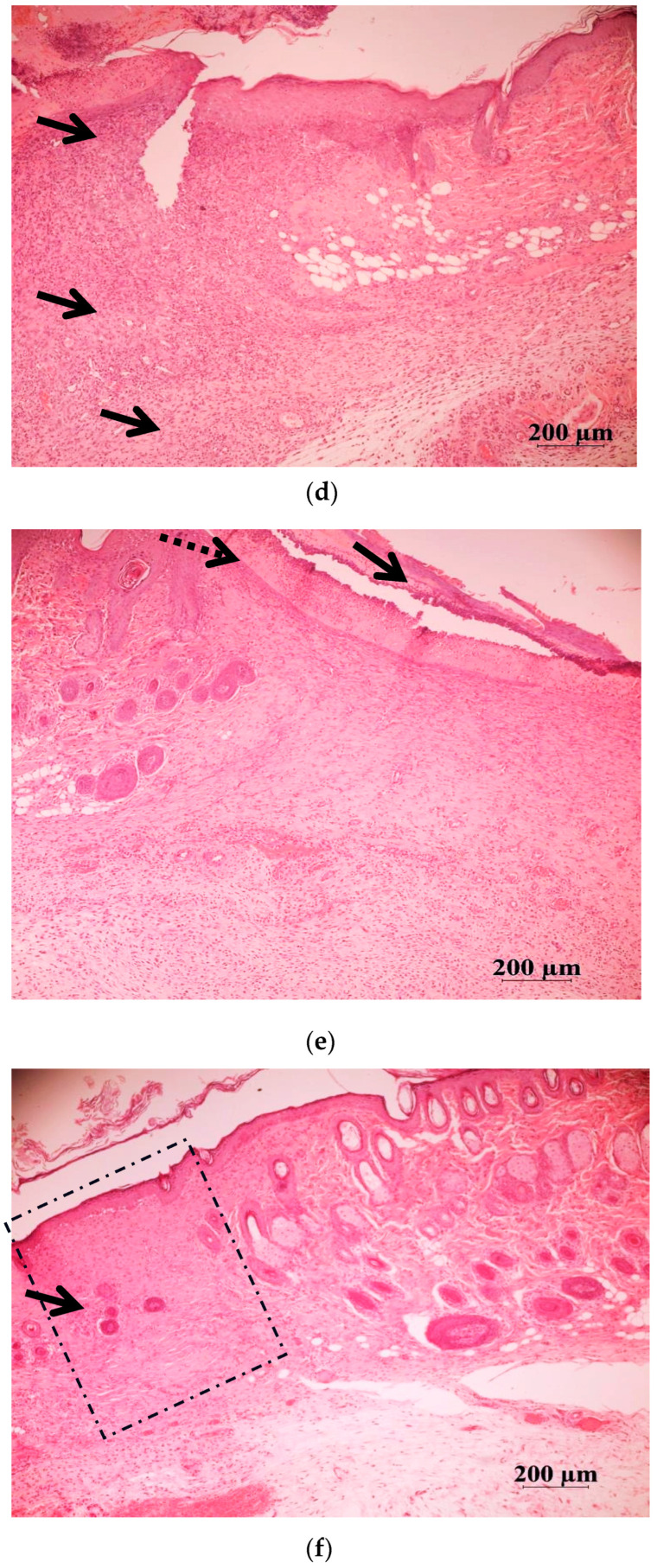
General histological picture in the skin defects at days 5 and 10 of the wound healing. Histological sections of the skin of rats from group O1 (treated with Hestatin 10) and O7 (placebo) in the area of healing defects were stained with hematoxylin and eosin. (**a**–**c**) group O1 (Hestatin 10); (**d**–**f**) group O7 (placebo). The scale segment is 200 µm, magnification ×50. (**a**,**d**) Day 5 of the wound process of the skin: the acute phase of inflammation in the wound area, with diffuse inflammatory infiltration (arrows); (**b**) on the 10th day, the wound defect is covered with a newly formed epidermis (arrows); the inflammatory infiltrate persists mainly subepithelially (dashed arrow); (**e**) the wound is covered with a scab (arrow), under which a thin layer of epidermis is formed (dashed arrow); the inflammatory process is noted both in the dermis and in the hypodermis, and the vessels are full-blooded (10th day); (**c**) on the 14th day, the skin layers in the wound area are fully formed and correspond to the normal structure; there are no signs of inflammation, the hair follicles are densely distributed, and they are in the stage of active growth (arrow); (**f**) the wound is covered with a thin layer of epidermis, and full-blooded vessels and slightly pronounced infiltration support a pro-inflammatory background, with single hair follicles (arrow), (14th day). The healing defect is marked with a dotted square.

**Figure 4:**
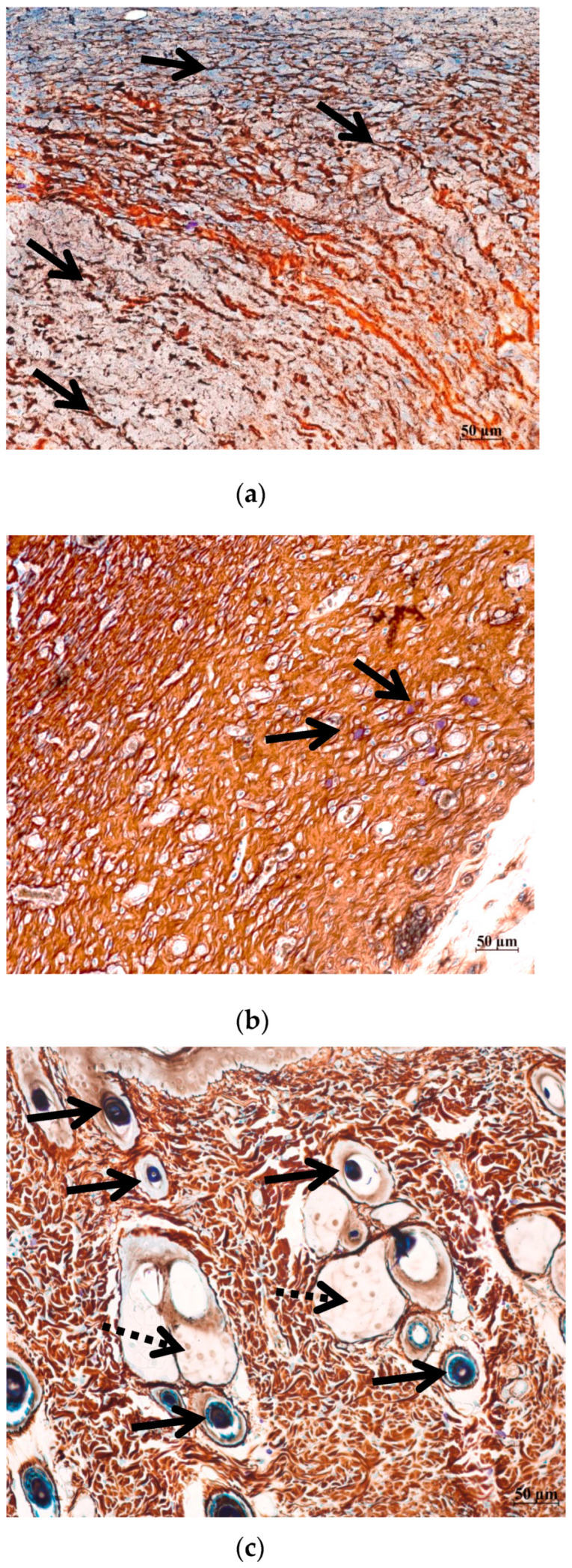

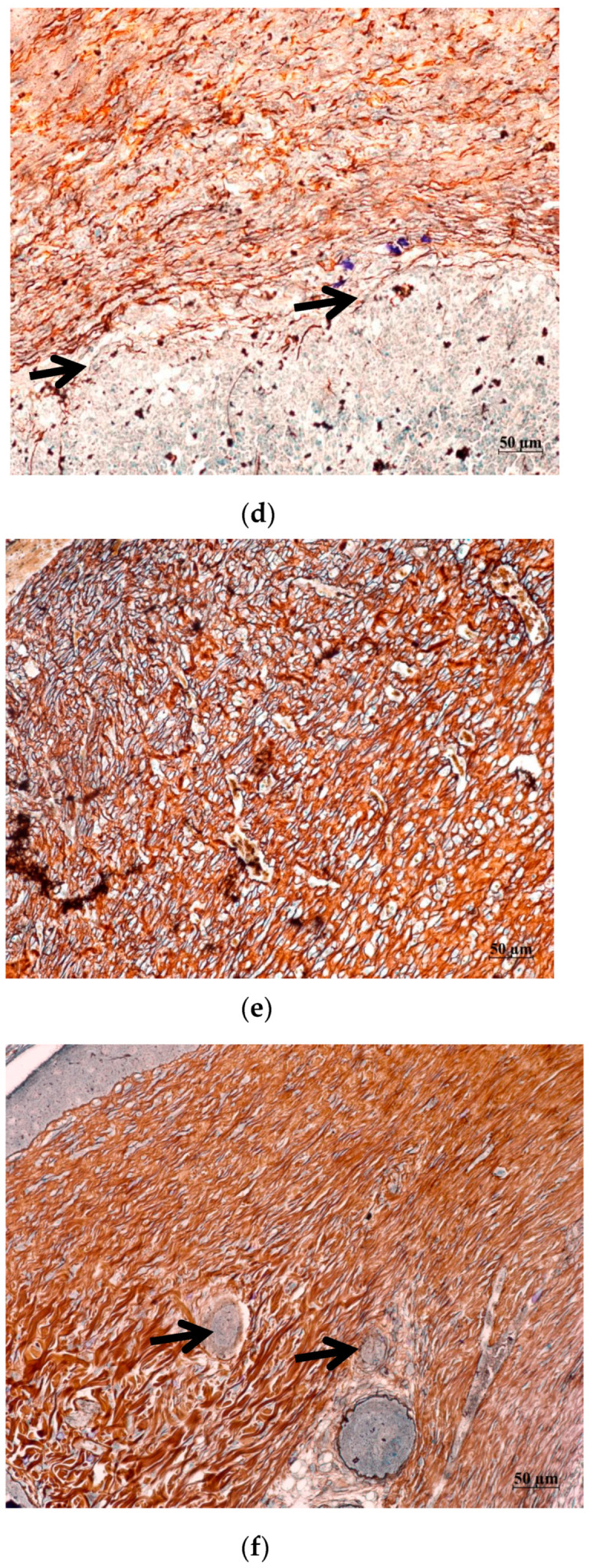
Remodeling of the fibrous component of the skin in different phases of the wound healing ((**a**,**d**) day 5; (**b**,**e**) day 10; (**c**,**f**) day 14). Histological sections of the skin of rats from group O1 (Hestatin 10) and group O7 (Placebo) in the area of healing defects were stained by a combined staining method with ammonium silver and toluidine blue solutions [13]. The scale segment is 50 µm, magnification 200×. (**a**–**c**) Group O1 (Hestatin 10); (**d**–**f**) group O7 (placebo). (**a**) Group O1, day 5—dermis of the skin in the wound area, reticular fibers (indicate black staining, arrows) form a fibrous framework, gradually replaced with few thick collagen fibers (indicate brown staining); (**b**) group O1, day 10—collagen fibers of an ordered orientation predominate over reticular fibers, and MCs are localized in foci (arrows); (**c**) group O1, day 14—the fibrous framework of the dermis of the skin in the wound area corresponds to the normal structure of the skin, many hair follicles are in the anagen phase (arrows), (active growth phase), and glandular skin structures are formed (dashed arrow); (**d**) group O7, day 5—few reticular fibers of the dermis (arrows); (**e**) group O7, day 10—the dermis is composed with scattered reticular fibers, collagen fibers are found in the hypodermic region; (**f**) group O7, day 14—the fibrous components of the dermis have a tightly ordered structure, and single hair follicles in the telogen phase (resting) occur (arrows).

**Figure 5:**
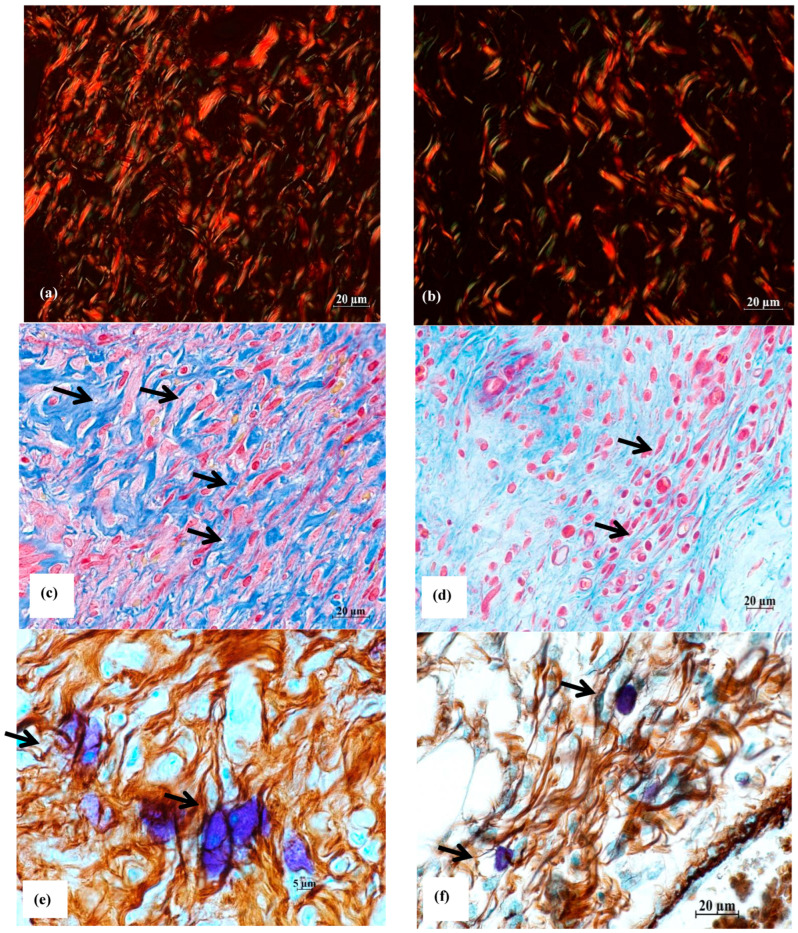
Reparative fibrillogenesis in the skin on day 5 of the wound healing. Histological sections of the skin of rats from group O1 (Hestatin 10) and group O7 (placebo) in the area of healing defects were stained by picrosirius red, picro-Mallory, and by the combined staining method with ammonium silver and toluidine blue solutions [13]. The scale segments are (**a**–**d**,**f**) 20 µm and (**e**) 5 µm; magnification ×400. (**a**) picrosirius red, polarization microscopy, group O1—formation of the connective tissue matrix of the dermis of the skin in the wound area, type III collagen fibers are stained with green, and type I collagen fibers are shown in red; (**b**) picrosirius red, polarization microscopy, group O7—a few thin, short fibers in the wound area, mainly represented by type III collagen (stained with green); (**c**) picro-Mallory, group O1—Hestatin 10 activates cellular representatives of the fibroblastic differon of the dermis of the skin in the wound area; (**d**) picro-Mallory, group O7—a few representatives of fibroblastic differon in the dermis; (**e**) combined impregnation staining with ammonium silver solution and toluidine blue, group O1—MC accumulation with signs of degranulation in the cellular structures of newly formed fibers of the dermis in the wound area; (**f**) combined impregnation staining with ammonium silver solution and toluidine blue, group O7—MCs with varying degranulation activity in colocalization with reticular (argyrophilic, black-colored) fibers in the wound area, thin reticular fibers in black, shades of brown—collagen fibers. The cellular representatives of fibroblastic differon are shown with arrows.

Currently, there is no commonly acknowledged model in the literature that provides a detailed description of the role of MCs, macrophages, fibroblasts, fibroblastic differentiation components, and myofibroblasts in the process of wound healing, scar formation, and restoration of the natural structure of the dermis, epidermis, and the basal membrane that separates them. Nevertheless, the significant contribution of all these cell types to the specific wound-healing mechanism is undeniable. As a result, on the 5th, 10th, and 14th days after the wound was created, two animals from each group were removed from the experiment to undergo histological analysis for the presence of reticular and collagen fibers in the area of the defect, as well as for the presence of MCs around and inside the wound (Figure 3 and Figure 4). The MC content with signs of degranulation was assessed separately (Figure 5).

For each defect, 10 visual fields were chosen, with the total length of the diagonal ranging from 15% to 30% of the defect’s perimeter. Consequently, the total area of the histologically examined dermis around the defect provided a representative sample, allowing conclusions to be drawn regarding the content of the analyzed components. For the analysis of reticular and collagen fibers, 20 sections per sample were used to ensure a comprehensive evaluation, with the sum of the diagonal lengths of these sections amounting to 5.3 mm. This encompassed 6.7% of the length of the defect perimeter on the first day of the experiment. In the case of MC analysis, 10 sections from the area under the bottom of the wound and 10 sections from the area on the perimeter of the wound were used, which provided the same coverage as in the case of calculating the proportion of fibers.

**Figure 6:**
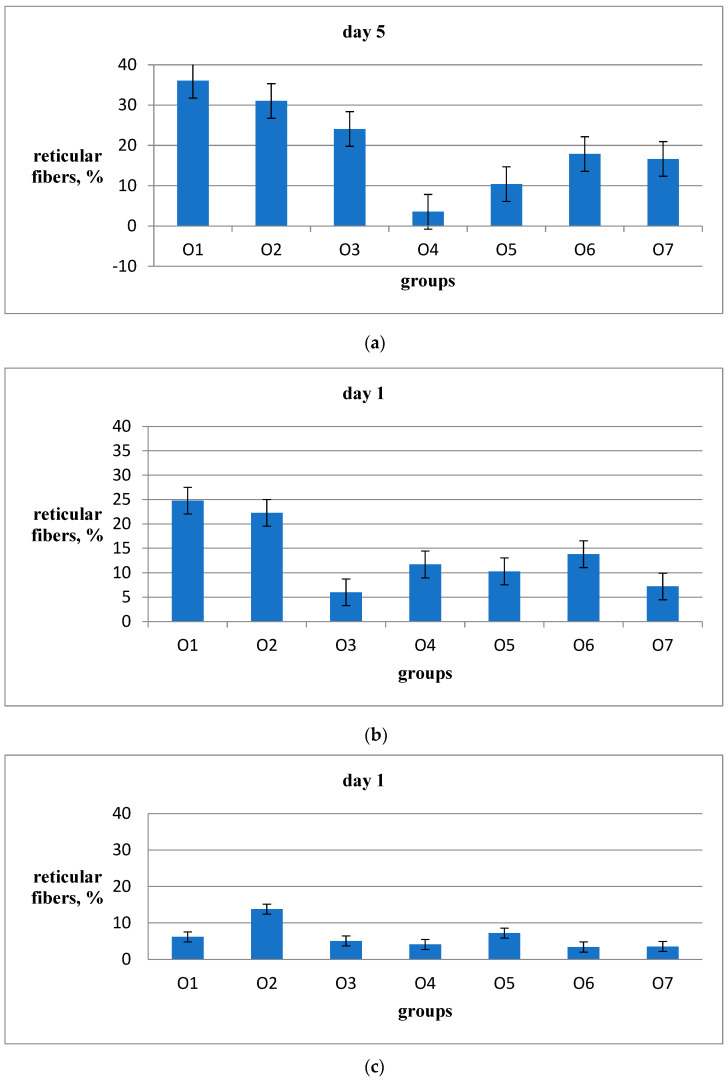
The experiment measured the presence of reticular fibers in wounds on the 5th (**a**), 10th (**b**), and 14th days (**c**). The percentage of the field of view area was calculated using a combined staining method with ammonium silver and toluidine blue solutions. The average values were obtained from 20 fields of view from 2 animals in each group. The vertical bars in the data represent the standard error of the average value for each group on the specified date. The vertical bars above the columns show the standard error for each group.

The proportion of dermis volume attributed to the reticular fibers is shown in Figure 6. On the fifth day of the experiment, this proportion increased from 18% in the placebo group O7 to 36% in the Hestatin 10 group O1. This indicates a significant impact of cobalt polyacrylate on accelerating the temporary connective tissue matrix synthesis. A somewhat smaller but still significant effect was observed in the O2 Hestatin 200 group, with the proportion of reticular fibers increasing to 31% of the dermis volume. Table 4 also shows significant differences between the placebo group O7 and PAA group O3 on the 5th day of the experiment. Meanwhile, the use of emulsion wax (group O4) and, to a lesser extent, resveratrol delivered with emulsion wax (group O5), resulted in a noticeable reduction in the proportion of reticular fibers compared to the placebo group (reduced by up to 4%). On the fifth day of the experiment, group O6 (Bepanthen) showed no significant differences from the placebo group O7. Therefore, the quantitative evidence of the wound-healing efficacy of Hestatin 10 and Hestatin 200 suggests that their effects may stem from non-conventional molecular mechanisms different from those of known wound-healing agents.

The analysis of reticular fibers in the dermis around the wound on the 10th day of the experiment showed that the O1 (25%) and O2 (22%) groups had higher levels compared to the 7% in the placebo group (O7). Meanwhile, the O3 (PAA) group did not differ from O7 or O4. However, the O5 and O6 groups showed significantly higher levels (Table 4). On the 14th day of the experiment, the O2 group (Hestatin 200) had the highest content of reticular fibers (14%), which was about four times higher than the placebo group (3.5%). The O1 (Hestatin 10) and O5 (resveratrol) groups showed a slight increase over the placebo group (6%), while the other groups, including O4 (resveratrol) and O6 (Bepanthen), exhibited no significant difference compared to the placebo group (Table 4).

**Figure 7:**
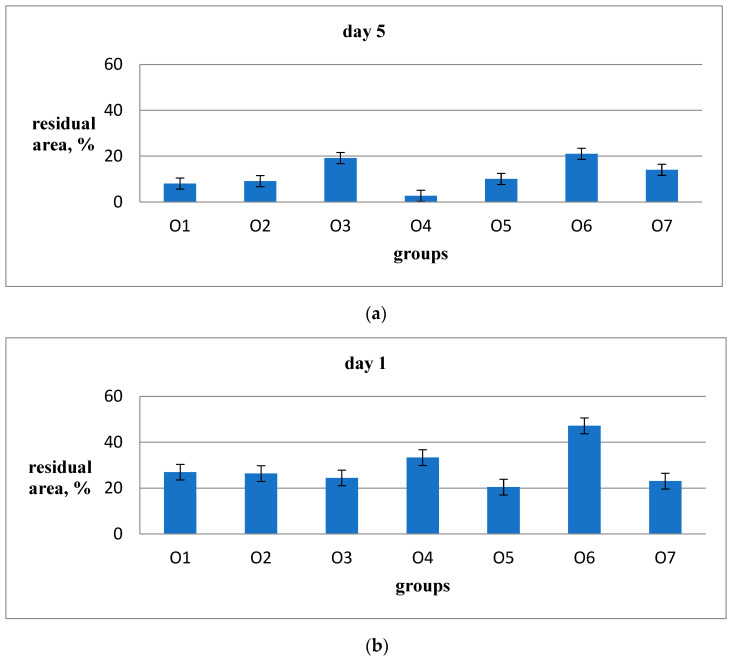

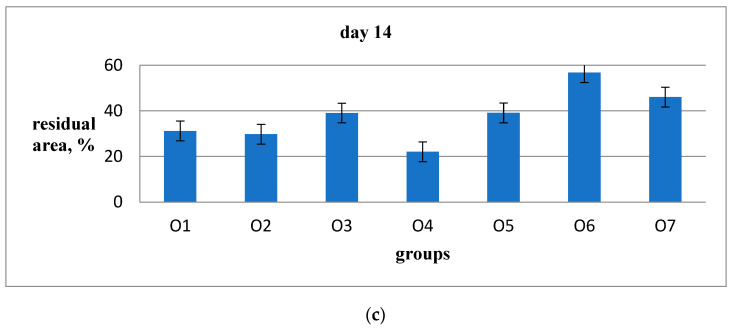
The percentage of the area with collagen fibers was measured on the 5th (**a**), 10th (**b**), and 14th (**c**) days of the experiment. This measurement was normalized to the field of view using a combined staining method with a solution of ammonium silver and toluidine blue. The average values were calculated based on 20 fields of view from 2 animals in each group, and the standard error of the average value for the group on each specified date is shown with vertical bars.

The analysis of collagen fiber content indicated an inhibition of synthesis when exposed to Hestatin 10 and Hestatin 200 compared to the placebo group O7 (Figure 7).

The goal was to confirm whether there were statistically significant differences between animal groups in terms of the content of collagen fibers in wounds on the 5th, 10th, and 14th days (Table 5).

These data were collected on the 5th, 10th, and 14th days of the experiment. On the 14th day, there was a significant difference of about 30% in the O1 and O2 groups versus 45% in the O7 group. It is important to note that the use of PAA (group O3) and resveratrol (O5) had a minimal effect on the collagen fiber content throughout the experiment. The use of Bepanthen ointment notably increased the content of this component, reaching 60% on the 14th day compared to 40% in the placebo group (Table 5). The use of emulsion wax also considerably slowed down the formation of collagen fibers, with levels at 30% on the 10th day and 22% on the 14th day. These findings suggest that Hestatin 10 and especially Hestatin 200 promote the remodeling of the collagen matrix, allowing it to restore the native structure of the dermis and epidermis. Additionally, on the 14th day of the experiment, animals from groups O1 and O2 showed the maximum number of hair follicles compared to other groups, while group O6 (Bepanthen) had a lower number than the placebo group (O7). On the other hand, Bepanthen, a commercial drug, accelerates collagen synthesis compared to a placebo, but it causes the formation of scar tissue instead of the fully recovered dermis and epidermis. The use of resveratrol accelerates the synthesis of reticular fibers, which positively affects the restoration of the native structure of the dermis and epidermis, but it does not contribute to the breakdown of synthesized collagen fibers, an essential factor for wound healing in the final phase.

**Figure 8:**
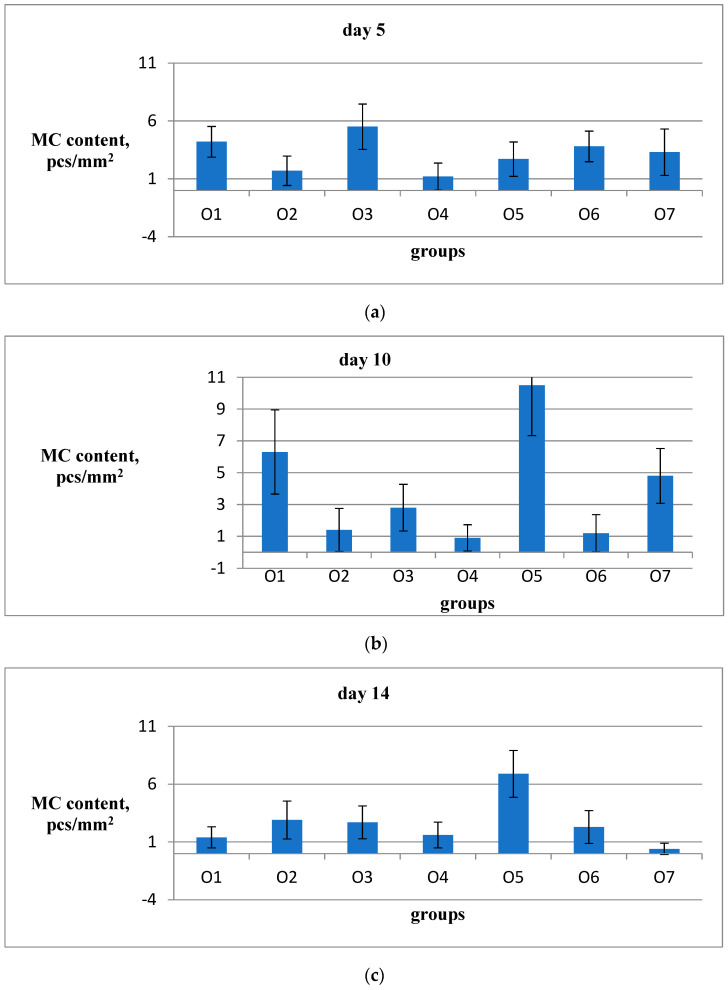
MC number in the wound bottom on the 5th (**a**), 10th (**b**), and 14th (**c**) days is shown. The results are presented in pcs/mm^2^ using a combined staining method with ammonium silver and toluidine blue. For each group, the average value was calculated based on 20 fields of view from 2 animals. The standard error of the average value for each group on the specified date is represented by the vertical bars.

The analysis of MC content in the wound bottom on the fifth day of the experiment, after staining with a combined method using toluidine blue, revealed the following results. In the O3 (PAA) group, there was a noticeable increase in MC content compared to the placebo O7 group (as shown in Figure 8a). In the O2 (Hestatin 200) and O4 (emulsion wax) groups, there was a decrease in MC content compared to the placebo group. However, the O1 (Hestatin 10), O5 (resveratrol), and O6 (Bepanthen) groups did not show significant differences in MC content in the wound bottom compared to the placebo group on the fifth day of the experiment (as indicated in Table 6).

During the 10th day of the experiment (Figure 8b), it was observed that there was a significant excess of MCs in the wound area in the O5 (resveratrol) group compared to the placebo. However, in the remaining groups O2, O3, O4, and O6, there was a decrease in the content of MCs in the wound area compared to the placebo group. This effect was especially pronounced in the groups O4 (emulsion wax) and O6 (Bepanthen). The group O1 (Hestatin 10) showed no significant differences in MC content in the wound bottom compared to the placebo on the 10th day of the experiment (Table 6). On the 14th day of the experiment (Figure 8c), a significant excess of MC in the wound bottom compared to the placebo was observed in all groups. This effect was greatest in the O5 (resveratrol) group and lowest in the O1 group (Hestatin 10).

From these observations, it can be concluded that in the early stages of wound healing, characterized by residual inflammation, Hestatin 10 and PAA can cause a significant increase in the number of MCs concentrating in the wound bottom. However, in the later phases of wound healing, these agents lose their effectiveness or even have a depressing effect on attracting MC to the wound area. On the other hand, resveratrol turns out to be the most effective stimulant for attracting MCs to the defect zone. Emulsion wax itself has a depressive effect on the formation of tissue under the wound bottom in all phases of wound healing. This effect, however, is mainly suppressed by resveratrol when injected with emulsion wax. Bepanthen has practically no effect on the behavior of MCs in the wound bottom in any phase of wound healing.

**Figure 9:**
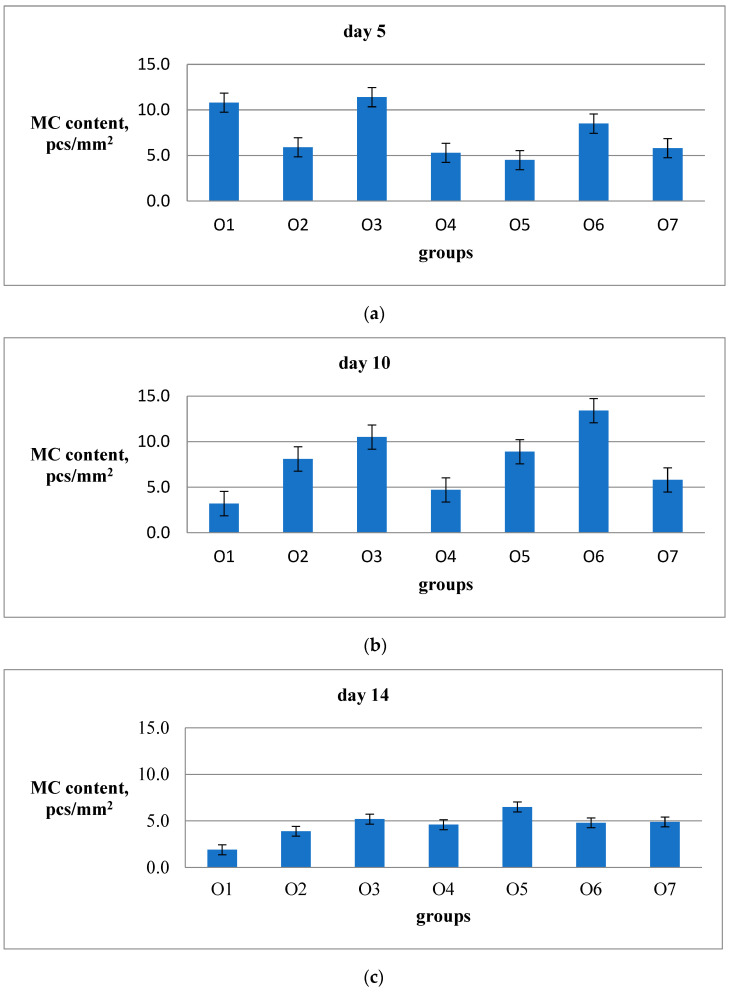
MC number in the normal skin at the wound perimeter on the 5th (**a**), 10th (**b**), and 14th (**c**) days is shown. The results are presented in pcs/mm^2^ using a combined staining method with ammonium silver and toluidine blue. For each group, the average value was calculated based on 20 fields of view from 2 animals. The standard error of the average value for each group on the specified date is represented by the vertical bars.

The impact of the drugs under study on the accumulation of MC in the undamaged skin around the healing wound demonstrates significantly different patterns compared to their effect on MC movement within the wound area (Figure 9). On the fifth day of the experiment, the O1 group (Hestatin 10), O3 group (PAA), and O6 group (Bepanthen) showed a higher accumulation of MCs at the wound perimeter compared to the placebo group. This observation is consistent with the effects of O1 and O3 observed in the wound bottom (Figure 9a). On the fifth day of the experiment, the O2, O4, and O5 groups showed no differences from the placebo group O7 in the MC content in undamaged skin around the healing wound (Table 7). However, the total MC content in the undamaged skin around the wound on the fifth day of the experiment was, on average, three times higher than that at the bottom of the wound. This suggests that the MCs located in this area may significantly influence the rate of wound healing.

During the 10th day of the experiment, it was observed that there was a notable increase in MC content in the intact dermis at the perimeter of the defect in groups O6 (Bepanthen) and O3 (PAA), compared to the placebo group. However, in group O1 (Hestatin 10), there was a significant decrease in MC content compared to the placebo group. The emulsion wax (O4) and Hestatin 200 (O2) did not show any significant effects on MC content at the wound perimeter compared to the placebo on the 10th day of the experiment (Table 7).

On the 14th day of the experiment, MC content on the perimeter of the healing defect in all groups became similar, except for group O1 (Hestatin 10), where it was significantly lower than the control. However, in groups O3 (PAA) and O5 (resveratrol), there was a significant excess of MC content at the perimeter of the healing defect compared to the placebo group. For groups O2 (Hestatin 200), O4 (emulsion wax), and O6 (Bepanthen), it was not possible to identify significant differences in MC content at the perimeter of the healing defect on the 14th day of the experiment (Table 7). The absolute MC content on the perimeter of the healing defect in all groups was, on average, twice as high as under the bottom of the wound during the same observation period.

After examining the samples, we assessed the MC content that underwent degranulation (as shown in Table 8). These cells displayed reduced staining intensity with toluidine blue. Due to the low absolute content of degranulating MC, it was challenging to statistically process the results and determine the reliability of the differences between the groups.

The data from Table 8 indicate that on the fifth day of the experiment, the use of PAA (group O3) resulted in the highest increase in the proportion of MC with signs of degranulation compared to the placebo. This aligns with the data showing the greatest effect of PAA on the total MC content at the bottom of the wound (Figure 8a) and its perimeter (Figure 9a) on the fifth day of the experiment. However, on the 10th and 14th days of the experiment, PAA lost its effectiveness in influencing the degranulation of MC, and resveratrol, delivered using emulsion wax, emerged as the most effective inducer of degranulation during this period. This corresponds with the impact of resveratrol on the total MC content at the bottom of the wound (Figure 8b,c) and partially on the perimeter of the wound (Figure 9c). It is important to note that the use of all tested drugs significantly increased the content of degranulated MCs compared to the placebo group, where this indicator was close to zero. These findings also align with the results of determining the total amount of MC in the wound area (Figure 8c) but not on its perimeter (Figure 9c).

## 3. Discussion

Both at home and during surgical operations, a person often faces skin damage that needs to be cured as quickly as possible. At the same time, a complete restoration of the natural structure of the skin, including the epidermis, dermis, sebaceous glands, and hair follicles, is desirable [14]. The wound-healing process is usually divided into four successive phases: hemostasis (formation of a fibrin clot), inflammation, primary closure of the defect (retraction), and remodeling [15].

At the stage of hemostasis, which usually lasts from several minutes to several hours, reducing the pain, minimizing blood loss, and preventing infection of the wound with pathogenic microorganisms are the priorities of emergency medical care [16]. Different long-term degradable exogenous agents, e.g., cellulose dressings (cotton wool and gauze), oxidized cellulose, chitin, and zeolite, are suggested for application onto the wound surface with regard to enhance the blood clotting [17,18,19]. For reducing iatrogenic bleeding during surgical operations, laser coagulation [20], plasma thermal coagulation [21], and cryogenic coagulation are suggested [22]. All these techniques lead to denaturation of proteins derived from the blood, fibrin clot, connective tissue matrix and skin cells. Although the listed methods of accelerating hemostasis reduce blood loss and accelerate the defect protection from the external environment, thus reducing the infiltration of infectious agents, the natural antimicrobial properties of the fibrin clot are violated, which creates refugees in the depth of the defect convenient for the survival and proliferation of pathogens. The presence of foreign agents on the surface of the defect enhances and lengthens the phase of inflammation and prevents the operation of the natural mechanisms of primary closure of the defect and wound contraction.

During the inflammation stage, which typically lasts from two to five days for small wound healing, a balance between wound disinfection mechanisms (especially the production of reactive oxygen species by cells) and the organization of the fibrin clot along with mechanically damaged remnants of the connective tissue matrix (CTM) is crucial for the overall duration of wound healing, enabling the transition to the replacement with a newly synthesized CTM [23]. Mast cells (MCs), originating from myeloid progenitor cells in the red bone marrow, accumulate in the depth of the derma surrounding the wound and within the wound itself, playing a vital role in this process [24]. MCs and the skin fibroblasts under their regulatory control serve as sources of important factors affecting the inflammatory phase, such as IL-6, platelet-derived growth factor (PDGF), and transforming growth factor beta (TGF-β) [25,26]. Furthermore, during MC degranulation, the release of contents from their basophilic granules leads to increased concentrations of heparin (an anticoagulant factor) and histamine (an inflammatory factor increasing capillary wall permeability) as well as chymase, tryptase, carboxypeptidase A, β-hexosaminidase, eicosanoids (thromboxane, prostaglandin D2, leukotriene C4) near the wound, among others, such as TNF-α, FGF, IL-4, SCF, and several pro-inflammatory chemokines [13,27]. Fibroblasts and macrophages release significant amounts of matrix metalloproteinases, particularly MMP3, contributing to the remodeling of the interstitial matrix. MCs regulate MMP9 activity, and there are reports that MC themselves produce this metalloproteinase [28]. Several factors have a significant impact on the process of remodeling the fibrous component of the dermis. This includes the balance of synthesis of different types of collagens and fibronectin by fibroblasts. MCs located near the defect’s surface can influence the behavior of other immune system cells, like macrophages, neutrophils, and lymphocytes. These cells engage in complex signaling interactions which, along with fibroblasts, determine the duration and effectiveness of the inflammatory phase. The transition from the inflammatory phase to the primary closure phase is characterized by the proliferation and differentiation of fibroblasts, as well as by the appearance of myofibroblasts in the defect area. During this period, reepithelization begins, and the network of vascular capillaries starts to restore itself. To effectively manage wounds during the inflammation phase, it is essential to use bactericidal agents to prevent infection and consider using agents that promote MC replacement with macrophages to reduce the duration of the inflammatory phase, minimize pain, and improve wound protection. Phenolic compounds of plant origin, such as stilbenes and flavonoids, are examples of such agents.

In the third phase of wound healing, which is the primary closure phase, several key processes take place. First, the fibrin clot is destroyed, and then there is intensive synthesis of reticular and collagen fibers along with the proliferation of fibroblasts. This phase also involves wound contraction due to the activity of myofibroblasts. In the case of small wounds, this phase typically lasts for about 7 days on average. It is during this phase that the difference between primary and secondary tension closure of the wound is most noticeable. In the case of primary tension closure, if the wound is not significantly infected, the defect area is mechanically reduced rapidly due to the activity of myofibroblasts, while the fibrin clot is simultaneously removed from the wound site as a result of macrophage activity. On the other hand, in the case of secondary tension closure, if the wound remains infected and suppurates for a prolonged period, it delays the onset and progress of interstitial matrix synthesis and fibroblast proliferation. Consequently, wound healing occurs through the formation of scar tissue, slowly replacing the fibrin clot and inflammatory infiltrate. This type of wound healing poses a risk of chronic (non-healing) wounds and keloid scars that may persist for life not only on the skin but also in the underlying tissues. Given this, it can be assumed that drugs stimulating the evacuation of debris from the wound, collagen, and reticulin synthesis, and preventing wound infection, could have a beneficial effect on the course and outcome of wound healing. Furthermore, remedies to prevent wound infection should be recommended at this stage. Simultaneous stimulation of collagen and reticulin synthesis and breakdown, which may seem contradictory, actually helps to accurately lay the main bundles of collagen, which is crucial for restoring the native skin structure. When collagen synthesis processes predominate over decay, it leads to the stabilization of randomly arranged bundles, resulting in the formation of a stable scar. This type of scar cannot be replaced by the fully recovered dermis containing sebaceous glands and hair follicles and is delimited from the epidermis by the basement membrane.

In the fourth and final phase of wound healing, also known as the remodeling phase, which typically lasts about 7 days, the processes of mass proliferation of fibroblasts and collagen synthesis are suppressed. Instead, accelerated breakdown of collagen and reticulin becomes critical for the outcome and speed of wound healing [29]. The risk of infection during this phase is very low because the wound is completely isolated from the external environment. Therefore, the medication used on the wound should promote accelerated collagen remodeling, which is essential for the formation of the basement membrane between the dermis and epidermis, hair follicles, sebaceous and sweat glands, and vascular capillaries, and restoring the normal healthy skin microbiome [30].

Dexpanthenol, a right-rotating alcoholic derivative of pantothenic acid [2] along with various phenolic compounds of plant origin, such as stilbenes and flavonoids, are currently being used or tested to accelerate wound healing [31]. Resveratrol is one of these compounds [8]. Additionally, lipoic acid [32] and folic acid [33] are suggested for this purpose. Despite numerous reports on the effectiveness of these compounds in accelerating wound healing, information about the mechanisms of their action on fibroblasts, keratinocytes, and other cells of the immune system under these conditions is limited and scattered. Furthermore, the potential negative effects of using these substances, such as the risk of chronic wounds and scar formation due to excessive collagen synthesis and insufficient MMP activity, are not widely discussed. There is also insufficient attention given to adjusting the goals of medical care as the various phases of wound healing progress.

Hestatin was previously suggested as a candidate hemostatic agent with wound-healing properties; however, an accurate report of its wound-healing mechanism has not been described [1]. To our best knowledge, in the world market, there are currently no commercially available hemostatic agents with wound-healing properties.

Resveratrol is a commonly acknowledged substance combining wound-healing and antimicrobial properties [8]. For this reason, it was chosen as a reference object for Hestatin in addition to Bepanthen. Several methods for resveratrol synthesis, including Pfitzner–Moffatt oxidation, Wittig–Horner condensation, and Mizoroki–Heck, Perkin, and Wittig reactions, have been described [34]. We have developed an original method for resveratrol synthesis, enabling us to obtain a prototype weighing more than 200 g with a purity of more than 99.8% according to NMR spectroscopy data (see Materials and Methods section). The synthesized resveratrol sample, in combination with emulsion wax, was used as a comparison sample during tests of Hestatin 10 and Hestatin 200, which are described in our previous article as hemostatic agents with antimicrobial activity. The issue of the development of optimal means of delivering resveratrol and other plant polyphenols is systematically discussed in the scientific literature and is not considered fully resolved [35]. We monitored the physiological activity of the emulsion wax used as a carrier for resveratrol delivery when applied to sterile wounds. Acute toxicity tests of Hestatin 10, conducted before the main experiment, showed its complete safety when administered intragastrically at a dosage of 5 g/kg of body weight. This makes it possible to consider this substance completely safe for external use in wound treatment. Tests of the effects of Hestatin 10 and Hestatin 200 on the rate of wound healing showed that Hestatin 10, but not Hestatin 200, can significantly accelerate the process of wound closure compared to the placebo and comparison preps, namely resveratrol delivered using emulsion wax and Bepanthen ointment in combination with Levomecol. Visual observation of the condition of wounds shows that Hestatin 10, being a hydrophilic substance, penetrates well into the forming defect and significantly accelerates the formation and compression of the fibrin clot. The use of Hestatin 10 has shown to reduce the duration of the inflammation phase and alleviate pain in animals. This results in calmer behavior in animals receiving Hestatin 10 compared to other drugs. On the other hand, hydrophobic preparations, such as Bepanthen, emulsion wax, and resveratrol, delivered using the emulsion wax, have been found to cause the formation of exudate on the surface of a fibrin clot compared to a placebo. Histological analysis of healing defects confirms that Hestatin 10, Hestatin 200, and PAA10 significantly speed up the formation of reticular fibers on the fifth day of the experiment, while suppressing the formation of collagen fibers. In the later stages of wound healing (10th and 14th days), the increased content of reticular fibers compared to the placebo group remains, although the difference between the groups gradually decreases. There is a slightly lower proportion of collagen fibers by the end of the wound healing period compared to the placebo group.

Additionally, the use of Hestatin 10 has a positive effect on the restoration of dermal vascularization, the formation of hair follicles, and the basal membrane separating the derma and epidermis. In contrast, the Bepanthen ointment suppresses the synthesis of reticular fibers and accelerates collagen synthesis from the initial phase of wound healing, resulting in the formation of coarse scar tissue devoid of blood vessels, hair follicles, and the separation of the derma and the epidermis. As for resveratrol, it has a positive effect in the later phases of wound healing, as it stimulates the remodeling of the collagen matrix and the partial restoration of the native structure of the derma and the epidermis. The analysis of the effect of the tested drugs on the MC content in the defect area shows that in the early stages of wound healing, Hestatin 10 stimulates their influx into the wound area from its perimeter. The use of Hestatin 10 has been shown to reduce the duration of the inflammation phase and alleviate pain in animals. This results in calmer behavior in animals receiving Hestatin 10 compared to other drugs. On the other hand, hydrophobic preparations, such as Bepanthen, emulsion wax, and resveratrol, delivered using emulsion wax, have been found to cause the formation of exudate on the surface of a fibrin clot compared to a placebo. Histological analysis of healing defects confirms that Hestatin 10, Hestatin 200, and PAA10 significantly speed up the formation of reticular fibers on the 5th day of the experiment, while suppressing the formation of collagen fibers. In the later stages of wound healing (10th and 14th days), the increased content of the reticular fibers compared to the placebo group remains, although the difference between the groups gradually decreases.

There is a slightly lower proportion of collagen fibers by the end of the wound healing period compared to the placebo group. Additionally, the use of Hestatin 10 has a positive effect on the restoration of dermal vascularization, the formation of hair follicles, and the basal membrane separating the derma and epidermis. In contrast, Bepanthen ointment suppresses the synthesis of reticular fibers and accelerates collagen synthesis from the initial phase of wound healing, resulting in the formation of coarse scar tissue devoid of blood vessels and hair follicles, and in the separation of the derma and the epidermis. As for resveratrol, it has a positive effect in the later phases of wound healing, as it stimulates the remodeling of the collagen matrix and the partial restoration of the native structure of the derma and the epidermis. The analysis of the effect of the tested drugs on MC content in the defect area shows that in the early stages of wound healing, Hestatin 10 stimulates their influx into the wound area from its perimeter.

## 4. Materials and Methods

### 4.1. Drugs for Testing

The Hestatin preparations were obtained from PAA variants with molecular weights of 9.356 and 184.631 Da, as detailed in Shibaeva et al., 2024 [1]. To remove weakly salt-bound Co^3+^ ions and hydrochloric acid, long-term dialysis was used. The preparations were then filtered for sterilization using a 22 nm pore diameter membrane and brought to a concentration of 10 g/L with sterile deionized water. PAA, with an average molecular weight of 9.356 Da, was purified by reverse osmosis and had a dry matter concentration of 10 g/L and a pH of 5.1. This PAA was previously used in the synthesis of Hestatin 10 for comparison.

The chemicals were purchased from commercial sources (Sigma-Aldrich, St. Louis, MO, USA) and used as received. All solvents were purchased from ALDOSA LLC, Moscow, Russia. A resveratrol preparation was synthesized using a modified technique involving four stages and purification of the sample according to the original method. The main stages followed previously described methods [9]. To improve the synthesis, the second stage was modified by using trimethylphosphite instead of triethylphosphite, resulting in a significant decrease in the reaction time and an increase in yield. Additionally, a method for purifying the final resveratrol preparation from traces of impurities was developed. This involved converting it to a tris-acetyl derivative with acetic anhydride and hydrolyzing the resulting product with catalytic amounts of sodium methylate. Commercially available reagents were used as starting materials, namely 3,5-dimethoxytoluene (CAS 4179-19-5) and 4-methoxybenzaldehyde (CAS 123-11-5).

The first stage of the process involved the bromination of 3,5-dimethoxytoluene (CAS 4179-19-5) with N-bromosuccinimide in absolute acetonitrile to form 1-(bromomethyl)-3,5-dimethoxybenzene with a yield of 91% (Formula (3)). The purity of the resulting product, as determined by 1H NMR spectroscopy, was above 96%. The detailed procedure for this stage of synthesis is described in a previous study [9].


(3)

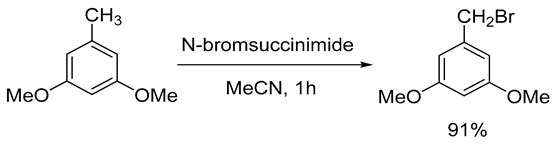



The second stage of the process involved the Arbuzov–Michaelis phosphorylation of 1-(bromomethyl)-3,5-dimethoxybenzene with trimethylphosphite at 120 °C for 15 min without a solvent (Formula (4)). This resulted in the formation of dimethyl (3,5-dimethoxyphenyl)phosphonate with a yield of 93%. The purity of the resulting product, determined by 1H NMR spectroscopy, was above 96%. The detailed procedure for carrying out this stage of synthesis has been described previously [36].


(4)

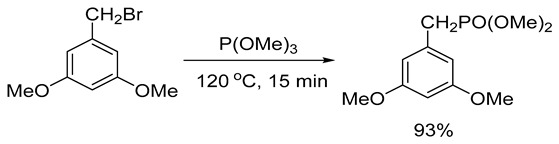



In the third stage of the process, diethyl (3,5-dimethoxyphenyl)phosphonate was condensed with 4-methoxybenzaldehyde (CAS 123-11-5) in tetrahydrofuran. This reaction took place at room temperature for 6 h in the presence of potassium hydroxide and the interfacial catalyst 18-crown-6 in methylene chloride (Formula (5)). The product formed was (E)-1,3-dimethoxy-5-(4-methoxystyryl)benzene, with a yield of 94% and a purity above 96% as determined by 1H NMR spectroscopy. The detailed procedure for this stage of the synthesis has been previously described [37].


(5)

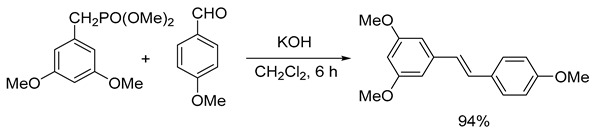



In the fourth stage, (E)-1,3-dimethoxy-5-(4-methoxystyryl)benzene was demethylated with boron tribromide in methylene chloride at −78 °C to form (E)-5-(4-hydroxystyryl)benzene-1,3-diol (resveratrol) with a yield of 92% (Formula (6)). The detailed procedure for this stage of the synthesis has also been described previously [8].


(6)

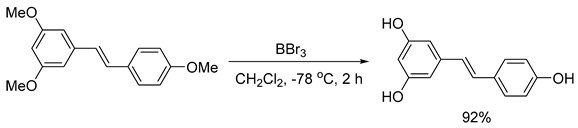



The pure resveratrol was dissolved in dimethyl sulfoxide (Dimexide, Rozlexfarm LLC, Russia) at a concentration of 250 g/L. The resulting concentrated solution was then used to prepare the final composition, which included Dimexide 40 g/L, polyvinylpyrrolidone K-90 (Orgpolymersynthesis LLC, Moscow, Russia) (200 g/L), emulsifier Olivem-1000 (Hallstar Beauty, USA) (120 g/L), and the synthesized resveratrol (10 g/L). Additionally, a control solution of emulsion wax without an active ingredient was prepared in parallel, with the following composition: Dimexide (Rozlexpharm LLC, Russia) (4%), polyvinylpyrrolidone K-90 (Orgpolymersynthesis LLC, Russia) (20%), and Olivem-1000 emulsifier (stearyl alcohol, a mixture of fatty acids; Hallstar Beauty, USA) (12%).

Bepanthen ointment for external use at a 5% concentration in an aluminum tube of 100 g is manufactured by GP Grenzach Productions GmbH (Germany) and was used for comparison. An antibacterial ointment for external use, Levomekol (40 mg/g of chloramphenicol as an active substance; Nizhpharm JSC, Nizhny Novgorod, Russia), was also used alongside Bepanthen.

### 4.2. Animals and Their Care

We performed experiments to study wound-healing activity in 42 male Wistar rats weighing between 180 and 210 g. These rats were obtained from the Stolbovaya branch NCBMT FMBA Institution (Russia). To ensure a fair trial, we randomized the animals by weight and sex using a method of pairs of similar characteristics and divided them into 7 groups, each containing 6 animals. The animal trials took place at the Research Institute of Experimental Biology and Medicine of Burdenko Voronezh State Medical University. The rats were kept in standard vivarium conditions, with 6 animals in each cage measuring 1 square meter, and subjected to a 12 h–12 h light–dark cycle. They were fed a granulated diet called Delta Feeds, in accordance with the standards for laboratory animals.

After being in the vivarium under standard conditions for 10 days, we determined the body weight and respiration rate and assessed the overall physiological state of the animals, including motor activity, behavior, feed and water consumption, reaction to stimuli, and the condition of visible mucous membranes, skin, subcutaneous adipose tissue, and hair.

In order to simulate a wound, the animals were first given inhalation anesthesia using isoflurane and a veterinary anesthesia station with a TES-3 vaporizer. A wound with an area of 50.24 mm^2^ was created using an 8 mm needle for a biopsy of the skin (an 8 mm Dermo-Punch (Medax, Poggio Rusco, Italy) on a pre-shaved area on the withers measuring 20–25 mm in diameter or 3–5 cm^2^ in square shape. The incision was made at the skin sampling site using the Dermo-Punch by rotating it clockwise and counterclockwise with slight pressure on the skin to penetrate the tissue. A strip of skin was then cut using tweezers and a scalpel. After creating the wound, the surface was treated with a 0.05% chlorhexidine solution (Akafarm LLC, Rostov Oblast, Russia). To standardize treatment conditions and prevent wound deformation, drying out, and contamination, a gauze bandage (sterile gauze (LLC KHBK VETEX, Moscow, Russia)) was applied over the wounds. On the 3rd day, the gauze bandage was removed, and the area of the primary wound was determined by photographing it with a ruler containing 1 mm divisions in the field of view by a high-resolution camera (Canon, Tokyo, Japan).

### 4.3. Wound Modelling, Treatment and Measurement of Residual Area

The animals in the experiment were monitored for 14 days to achieve the set goals and objectives. Throughout the experiment, the designated drugs and substances were applied to the wounds daily (see Table 3). The wound treatment involved the use of the synthetic resveratrol and emulsion wax preparations in conjunction with Levomecol ointment for external use (40 mg/g chloramphenicol; Nizhpharm JSC, Russia). In the experiment, the ointments were applied with disposable sterile medical spatulas in quantities of ≈0.5 g, depending on the degree of wound healing. The amount of ointment was taken so that when distributing the ointment over the wound surface, not only the scab area but also the adjacent tissues would be affected. The ointment distribution was controlled visually. The ointment was taken with a medical spatula and applied to the wound surface and adjacent tissues on the withers of the rat (the withers area was shaved to ensure contact of the ointment with the epidermis). Excess ointment, if any, was removed from the rat’s fur with the clean end of the spatula to prevent the ointment from entering the rat’s gastrointestinal tract when licking, and then the spatula with excess ointment was thrown away. A new sterile spatula was used for each treatment of each rat.

Daily monitoring included tracking integral indicators of the general condition and body weight of each animal. In addition, the residual area of the wound defect was determined with photo documentation, using a ruler with 1 mm increments in the field of view with a high-resolution camera (Canon, Japan). Visual indicators of the wound condition, such as the reaction to the application of the studied drugs, local signs of inflammation, and the condition of the scar, were recorded for each wound.

Furthermore, on the 5th, 10th, and 14th days, two animals were withdrawn from the experiment for histological analysis of the wound defect healing area.

### 4.4. Studies of the Acute Toxicity of the Studied Drugs in Mice with Intragastric Administration

The experiments involved white mice of both sexes weighing 22 ± 2 g. The animals were given Hestatin 10 and a comparison medicine called Bepanthen in the form of a prepared suspension in 1% starch paste, using a metal probe with a smooth olive at the end, in a quantity of 0.5 mL, which is the maximum volume allowed for this type of animal and their body weight.

To prepare the suspensions of Hestatin 10 and Bepanthen, they were mixed with 1% starch gel to ensure maximum drug concentration and the ability to administer the suspensions through the probe for intragastric administration to mice (mechanical dispenser, one channel, maximum volume of 200 μL for applying solutions to the wound (DLAB Toppette, Beijing, China). This was achieved by dissolving 200 mg of the experimental preparation and 1 g of Bepanthen in 1 mL of 1% starch gel, which was equivalent to 200 mg of the active substance. On average, mice weighing 20 g were injected with 0.1 g of the test preparation and the comparison medicine, which corresponds to a dose of 5 g/kg. The equivalent human dose for a person weighing 70 kg would be approximately 28 g. The toxic effect of the drugs was assessed by monitoring the general condition of the animals and their survival rate (LD_50_). The number of surviving and deceased animals was recorded on the 3rd day after administration of the experimental samples, followed by observing the surviving animals for an additional 2 weeks. Based on the results obtained, LD_50_ was calculated according to the guidelines for conducting preclinical studies of medicines edited by A.N. Mironov [38].

### 4.5. Preparation of Tissue Sections and Their Histochemical Analysis

For a histological examination of the tissues surrounding the healing defect, two animals from each group were removed on the 5th, 10th, and 14th days after the start of the experiment. The animals were euthanized using an isoflurane inhalation anesthesia overdose. Skin samples with an 8 mm diameter were then excised using a veterinary anesthesia station with a TES-3 evaporator for further histological analysis. The excised skin samples were immediately fixed in 10% buffered neutral formalin, labeled according to the animal group, and prepared for standard sample preparation [13].

For histological staining, 4 µm thick sections were made from each sample and stained with hematoxylin and eosin for general microscopic analysis of both the wound area and the surrounding skin area with an average diagonal length of 265 ± 35 µm. The content of reticular and collagen fibers, as well as the mast cell share and their degranulation activity, were analyzed using a combined staining method, which involved silver impregnation and toluidine blue [13]. The sections were examined in polarized light after staining with picrosirius red (Picrosirius red kit, ab150681, Abcam, Cambridge, UK). Additionally, to visualize the fibroblastic components and identify newly formed vascular capillaries in the connective tissue, sections were stained using the picro-Mallory technique as described earlier [39].

Morphometric analysis involves measuring the ratio of reticular and collagen fibers in the wound area and perifocal area using a 20× lens (200× magnification, high-resolution camera (Canon, Japan)) for 10 fields of view for each area. Quantitative analysis was performed for each field of view using the ImageJ program with the photograph transferred to the RGB STACK format. The proportion of the area occupied by thin (reticular) fibers and thick (collagen) fibers with a diameter of more than 1 µm was calculated from 10 fields of view for each area.

In addition, mast cell quantification and assessment of their degranulation activity were conducted in both the wound area and perifocal area across 10 fields of vision. The data are presented as the total number of cells in all fields of vision, with a calculation of the relative mast cell content per 1 mm^2^ of the tissue slice area.

### 4.6. Statistical Analysis

The results of the sample examination were recorded in the primary report tables. Statistical processing of data on measuring the rate of wound healing in groups was carried out as follows. The accuracy of measuring the area of wounds in rats by day was limited by the area of the scab. Under the scab, the wound may have been a significantly smaller size than the area of the scab. There was also variability in measuring the area of the scab due to the subjectivity of assessing the location of the edge. The reproducibility of determining the defect area using the same photo was found to be ± 7%. To reduce the error in processing each photo of the defect, 10 independent measurements of the area of a defect were carried out at each time point, using a ruler with divisions of 1 mm located in the frame for normalization. The following formula was used to calculate the required value:

The wound healing rate (V) = S (wound size in mm^2^)/t (healing time in days).

Wound healing rates were measured on the 5th, 10th, and 14th days, as it is difficult to measure the wound area at an earlier time due to inflammatory processes occurring in wounds blurring the wound boundary. The primary data set for the analysis included measurements taken by three independent observers in two to three repetitions using photographs. The wound was considered healed if the size of the residual defect was less than or equal to 1 mm^2^. After determining the moment when the residual area of the wound reached this value, the rate of decrease in the area of the wound from its initial area to zero was calculated. The rate of wound healing was expressed in mm^2^ per day.

The average values of the wound healing rate were checked to see if they were normally distributed. This allowed us to use the Student’s *t*-test to compare the experimental and control groups. The Mann–Whitney criterion, which is nonparametric, resulted in a higher error and could not be used to compare the results from the small groups of animals used in the experiment.

When testing the hypothesis regarding significant differences between animal groups in terms of parameters determined by histological methods (such as the proportion of reticular and collagen fibers in the field of view, MC content, as well as the number of MC with and without signs of degranulation per mm^2^ of the cut area), nonparametric Mann–Whitney analysis was used. Differences between the groups were considered significant if the Fisher parameter p did not exceed 0.05.

## Figures and Tables

**Table 1:** A comparison of the acute toxicity of Hestatin 10 and Bepanthen by GP Grenzach Productions GmbH (Germany) in mice following intragastric administration.

Animal Group	Dose g/kg	Animal Number			Lethality, %	LD_16_ mg/kg	LD_50_ mg/kg	LD_84_ mg/kg
		Total	Dead	Alive				
I	5	12	0	12	0	no	no	no
II	5	12	0	12	0	no	no	no

Note: Group I was treated with Hestatin 10, and Group II received Bepanthen by GP Grenzach Productions GmbH (Germany). LD values are given in mg per kg.

**Table 2:** Information about samples sent for the testing of wound-healing activity.

#	Group	pH	Name	Composition
1	O1	5.1	Hestatin 10	Cobalt polyacrylate purified by reverse osmosis derived from PAA with Mr = 10 kDa, a dry matter concentration 10 g/L, and a content of the tightly bound Co^3+^ ion 4.3% (*w*/*w*) of the dry matter
2	O2	5.1	Hestatin 200	Cobalt polyacrylate purified by reverse osmosis derived from PAA with Mr = 200 kDa, a dry matter concentration 10 g/L, and a content of tightly bound Co^3+^ ion 3.6% (*w*/*w*) of the dry matter
3	O3	5.1	PAA used for manufacturing Hestatin 10	Reverse osmosis-purified PAA with Mr = 10 kDa, and a dry matter concentration (10 g/L)
4	O4	5.0	Emulsion wax	Dimexide (LLC Rozlexfarm, Tverskaya, Russia) (4%), polyvinylpyrrolidone K-90 (Orgpolymersynthesis LLC, Russia) (20%), emulsifier Olivem-1000 (cetearyl alcohol, and a mixture of fatty acids; Hallstar Beauty, Chicago, IL, USA) (12%)
5	O5	5.0	Resveratrol delivered by emulsion wax	Dimexide (Rozlexpharm LLC, Russia) (40 g/L), polyvinylpyrrolidone K-90 (LLC Orgpolymersynthesis, Russia) (200 g/L), emulsifier Olivem-1000 (Hallstar Beauty, USA) (120 g/L), and synthetic resveratrol (10 g/L)
6	O6	5.6	Bepanthen ointment, a commercially available wound-healing drug derived from dexpanthenol, and levomecol ointment derived from chloramphenicol	Bepanthen ointment for external use (5%), aluminum tube (100 g): cardboard pack, code EAN 04250369504244, 4250369504244, 4601907000450, N N013984/01, valid from 05/19/2008, GP Grenzach Productions GmbH (Germany)
7	O7	5.3	Placebo	Deionized water

**Table 3:** The table shows the rate of wound healing in different animal groups in mm^2^ per day, based on the number of initial measurements of wound size. The rate of the wound healing was calculated by dividing the initial area of the wound by the number of days it took for complete healing. Complete healing was accounted for in three independent ways: (1) the day since which the residual area of the defect could not be measured; (2) the day since which the residual area of the defect was <1 mm^2^; (3) the day since which the residual area of the defect was <2 mm^2^. The initial wound square was measured on photos by three independent operators who delineated the area of the defect, trying to exclude the area of the scab covering healthy skin and extending beyond the edge of the wound. All the results measured in this way were averaged for each animal group, checked for the normality of the distribution, and the significance of the differences between each of the groups and the placebo O7 group was analyzed using the χ-square method (probability of coincidence is less than 5%).

Method of Registering the Completion of the Wound Healing	O1	O2	O3	O4	O5	O6	O7
Determining the day since which the residual area of the defect could not be measured	4.53	4.30	4.53	4.25	4.84	4.50	3.82
	3.63	5.50	4.16	4.54	4.53	5.73	4.07
	4.21	5.33	4.27	4.02	5.04	4.18	3.17
	4.93	3.15	4.49	3.64	5.95	4.48	4.14
	5.77	3.80	5.12	4.85	3.85	3.95	4.03
	5.80	4.81	5.06	4.89	5.04	5.39	4.29
	5.02	6.58	4.68	5.86	4.43	6.35	4.32
	5.36	4.85	4.51	5.31	4.32	5.11	4.24
	6.59	3.85	5.16	3.78	6.23	5.98	3.92
	6.51	4.32	5.84	4.97	5.22	4.51	4.88
Determining the day since which the residual area of the defect was <1 mm^2^	4.29	7.07	4.16	6.49	4.90	7.37	4.07
	5.41	7.46	5.50	4.75	5.96	5.10	4.36
	4.93	4.33	6.18	3.95	6.45	4.48	4.14
	6.49	4.64	6.25	5.73	5.56	4.67	4.70
	5.02	6.58	4.68	7.53	5.23	6.35	4.32
	6.13	5.55	5.80	5.31	4.68	5.68	4.77
	6.59	4.28	5.16	4.09	6.75	5.98	3.92
	7.44	4.80	6.49	5.87	5.80	4.89	4.88
Determining the day since which the residual area of the defect was <2 mm^2^	4.29	7.07	4.62	6.49	5.88	7.37	4.47
	4.29	7.07	4.62	6.49	5.88	7.37	4.47
	5.41	7.46	6.41	4.75	6.55	5.10	4.36
	5.54	4.95	6.18	4.31	7.04	4.48	5.32
	8.65	5.22	6.25	7.01	5.56	5.14	5.64
	5.02	7.52	5.20	7.53	5.23	7.25	4.76
	6.13	6.47	5.80	5.31	5.62	5.68	4.77
	6.59	4.28	6.45	5.46	7.37	5.98	5.04
	7.44	4.80	6.49	5.87	5.80	5.34	4.88
**Mean ± St.dev.**	5.63 ± 1.18	5.41 ± 1.30	5.34 ± 0.81	5.30 ± 1.12	5.55 ± 0.86	5.50 ± 1.01	4.44 ± 0.51
**Dispersion**	1.38	1.69	0.65	1.25	0.74	1.03	0.26
**t-criterion**	5.20 *	6.38 *	2.49 *	4.70 *	2.81 *	3.94 *	1.00

The “*” sign indicates the values of the Student’s *t*-test parameter that surpass the critical t-value. This shows a statistically significant difference in the average rate of wound healing in the group compared to the control (O7), with the probability of a random deviation in values not exceeding 5%.

**Table 4:** The results of the two-sample *t*-test, which was used to determine whether differences between experimental groups and the placebo group (O7) in terms of reticular fiber content on the 5th, 10th, and 14th days, were statistically significant. If the value of the two-sample *t*-test did not exceed 0.05, it was concluded that the hypothesis of random data matching was rejected, indicating a 5% probability of random matching.

Group #	Day 5	Day 10	Day 14
O1	0.00	0.00	0.00
O2	0.00	0.00	0.00
O3	0.00	0.19	0.02
O4	0.00	0.00	0.30
O5	0.00	0.00	0.00
O6	0.12	0.00	0.81

**Table 5:** The differences in the content of collagen fibers in wounds on the 5th, 10th, and 14th days. The values of the two-sample *t*-test when comparing each group with the placebo group (O7). If the value of the two-sample *t*-test did not exceed 0.05, the hypothesis of the random nature of the data match for the two samples was rejected, indicating a statistically significant difference (with a 5% probability of random matching in this case).

Group #	Day 5	Day 10	Day 14
O1	0.00	0.00	0.01
O2	0.00	0.00	0.00
O3	0.00	0.20	0.03
O4	0.00	0.00	0.31
O5	0.00	0.00	0.00
O6	0.12	0.00	0.81

**Table 6:** We tested the hypothesis of whether there were statistically significant differences between groups of animals in terms of MC content in the wound bottom on the 5th, 10th, and 14th days of the experiment. The table shows the values of the two-sample *t*-test when comparing each group with the placebo group (O7). If the value of the two-sample *t*-test did not exceed 0.05, it was considered that there were significant differences (with a 5% probability of random matching).

Group #	Day 5	Day 10	Day 14
O1	0.28	0.17	0.00
O2	0.06	0.00	0.00
O3	0.03	0.02	0.00
O4	0.01	0.00	0.01
O5	0.48	0.00	0.00
O6	0.54	0.00	0.00

**Table 7:** To test the hypothesis of significant differences between animal groups in terms of MC content in the normal skin around the wounds, a two-sample *t*-test was used to compare each group with the placebo group (O7) on the 5th, 10th, and 14th days of the experiment. A two-sample *t*-test value not exceeding 0.05 indicates a rejection of the hypothesis of random data matching, with a 5% probability of a random matching in this case.

Group #	Day 5	Day 10	Day 14
O1	0.00	0.00	0.00
O2	0.90	0.15	0.15
O3	0.00	0.00	0.00
O4	0.66	0.17	0.68
O5	0.16	0.00	0.00
O6	0.04	0.00	0.90

**Table 8:** MC content that underwent degranulation was measured in pcs per mm^2^ of section surface.

Group #	Day 5	Day 10	Day 14
O1	8.17 ± 2.11	11.23 ± 3.4	8.23 ± 3.11
O2	6.91 ± 2.23	5.32 ± 2.43	14.22 ± 1.91
O3	21.47 ± 3.44	9.31 ± 2.71	9.28 ± 2.55
O4	5.19 ± 2.32	3.78 ± 2.65	6.57 ± 1.76
O5	5.10 ± 2.56	32.85 ± 2.17	26.82 ± 2.43
O6	13.36 ± 3.04	5.65 ± 2.48	8.48 ± 2.59
O7	9.22 ± 2.22	18.12 ± 2.39	0.59 ± 0.76

## Data Availability

The study does not contain any new data or confidential data registered in accordance with scientific and ethical requirements.

## References

[1] Shibaeva A.V., Vasilieva A.P., Bokareva O.P., Samborsky S.A., Smirnova M.S., Shirshin K.K., Bogdanova E.S., Trubnikova E.V., Shevelev A.B. (2024). Synthesis of the cobalt polyacrylate (Hestatin) and testing of its haemostatic properties. BioNanoScience.

[2] Ebner F., Heller A., Rippke F., Tausch I. (2002). Topical Use of Dexpanthenol in Skin Disorders. Am. J. Clin. Dermatol..

[3] Youjun D., Huang Y., Lai Y., Ma Z., Wang X., Chen B., Ding X., Tan Q. (2023). Mechanisms of resveratrol against diabetic wound by network pharmacology and experimental validation. Ann. Med..

[4] Hou C.Y., Tain Y.L., Yu H.R., Huang L.T. (2019). The Effects of Resveratrol in the Treatment of Metabolic Syndrome. Int. J. Mol. Sci..

[5] Ahmad J., Ahamad J., Algahtani M.S., Garg A., Shahzad N., Ahmad M.Z., Imam S.S. (2024). Nanotechnology-mediated delivery of resveratrol as promising strategy to improve therapeutic efficacy in triple negative breast cancer (TNBC): Progress and promises. Expert Opin. Drug Deliv..

[6] Wang X., Liu C., Wang J., Tian Z. (2024). Resveratrol suppresses NSCLC cell growth, invasion and migration by mediating Wnt/β-catenin pathway via downregulating SIX4 and SPHK2. J. Chemother..

[7] Hashemi M., Aminzare M., Hassanzadazar H., Roohinejad S., Tahergorabi R., Bekhit A.E.A. (2023). Impact of sodium alginate-based film loaded with resveratrol and thymol on the shelf life of cooked sausage and the inoculated Listeria monocytogenes. Food Sci. Nutr..

[8] Shevelev A.B., La Porta N., Isakova E.P., Martens S., Biryukova Y.K., Belous A.S., Sivokhin D.A., Trubnikova E.V., Zylkova M.V., Belyakova A.V. (2020). In Vivo Antimicrobial and Wound-Healing Activity of Resveratrol, Dihydroquercetin, and Dihydromyricetin against Staphylococcus aureus, Pseudomonas aeruginosa, and Candida albicans. Pathogens.

[9] Martínez A.V., García J.I., Mayoral J.A. (2017). An expedient synthesis of resveratrol through a highly recoverable palladium catalyst. Tetrahedron.

[10] El-Deeb I.Y., Funakoshi T., Shimomoto Y., Matsubara R., Hayashi M. (2017). Dehydrogenative Formation of Resorcinol Derivatives Using Pd/C–Ethylene Catalytic System. J. Org. Chem..

[11] Jungong C.S., Novikov A.V. (2012). Practical Preparation of Resveratrol 3-O-β-D-Glucuronide. Synth. Communicat..

[12] Guiso M., Marra C., Farina A. (2002). A new efficient resveratrol synthesis. Tetrahedron Lett..

[13] Atiakshin D., Soboleva M., Nikityuk D., Alexeeva N., Klochkova S., Kostin A., Shishkina V., Buchwalow I., Tiemann M. (2023). Mast Cells in Regeneration of the Skin in Burn Wound with Special Emphasis on Molecular Hydrogen Effect. Pharmaceuticals.

[14] Baron J.M., Glatz M., Proksch E. (2020). Optimal Support of Wound Healing: New Insights. Dermatology.

[15] Childs D.R., Murthy A.S. (2017). Overview of Wound Healing and Management. Surg. Clin. N. Am..

[16] Reinke J.M., Sorg H. (2012). Wound repair and regeneration. Eur. Surg. Res..

[17] Li S., Wu X., Bai N., Ni J., Liu X., Mao W., Jin L., Xiang H., Fu H., Shou Q. (2023). Fabricating Oxidized Cellulose Sponge for Hemorrhage Control and Wound Healing. ACS Biomater. Sci. Eng..

[18] Gan C., Hu H., Meng Z., Ni J., Liu X., Mao W., Jin L., Xiang H., Fu H., Shou Q. (2023). Local Clays from China as Alternative Hemostatic Agents. Molecules.

[19] Su C., Cao Z., Liu J., Sun X., Qiu K., Mu Y., Cong X., Wang X., Chen X., Jia N. (2023). The hierarchical porous structures of diatom biosilica-based hemostat: From selective adsorption to rapid hemostasis. J. Colloid Interface Sci..

[20] Taniguchi Y., Sawada K., Yamada A., Mizutani K., Meinzer W., Iwata T., Izumi Y., Aoki A. (2021). Er:YAG Laser-Assisted Bone Regenerative Therapy for Implant Placement: A Case Series. Int. J. Periodontics Restor. Dent..

[21] Jain N., Mohan J.A., Ramita S., Kanchan S., Amandeep K., Meena S. (2023). Argon plasma coagulation therapy in hemorrhagic radiation proctitis following pelvic radiation in gynecological malignancies. J. Cancer Res. Ther..

[22] Fieb A., Gibler S., Mildenberger E., Urschitz M.S., Fauer A., Elflein H.M., Zepp F., Stoffelns B., Pfeiffer N., Schuster A.K. (2022). Anterior Chamber Angle in Adults Born Extremely, Very, and Moderately Preterm with and without Retinopathy of Prematurity-Results of the Gutenberg Prematurity Eye Study. Children.

[23] Wolfram D., Tzankov A., Pülzl P., Piza-Katzer H. (2009). Hypertrophic scars and keloids—A review of their pathophysiology, risk factors, and therapeutic management. Dermatol. Surg..

[24] Ribatti D., d’Amati A. (2023). Hematopoiesis and Mast Cell Development. Int. J. Mol. Sci..

[25] Slavin J. (1996). The role of cytokines in wound healing. J. Pathol..

[26] Turksen K. (2017). Wound Healing: Stem Cells Repair and Restorations, Basic and Clinical Aspects.

[27] Bayat A., McGrouther D.A., Ferguson M.W.J. (2003). Skin scarring. BMJ.

[28] Xu L., Cai Z., Yang F., Chen M. (2017). Activation-induced upregulation of MMP9 in mast cells is a positive feedback mediator for mast cell activation. Mol. Med. Rep..

[29] Chouhan D., Janani G., Chakraborty B., Nandi S.K., Mandal B.B. (2018). Functionalized PVA-silk blended nanofibrous mats promote diabetic wound healing via regulation of extracellular matrix and tissue remodeling. J. Tissue Eng. Regen. Med..

[30] Xu W., He M., Lu Q. (2024). Fibronectin connecting cell sheet based on click chemistry for wound repair. Adv. Sci..

[31] El-Sherbeni S.A., Negm W.A. (2023). The wound healing effect of botanicals and pure natural substances used in in vivo models. Inflammopharmacology.

[32] He D., Liao C., Li P., Liao X., Zhang S. (2024). Multifunctional photothermally responsive hydrogel as an effective whole-process management platform to accelerate chronic diabetic wound healing. Acta Biomater..

[33] Duman N., Duman R., Tosun M., Akıcı M., Göksel E., Gökçe B., Alagöz O. (2018). Topical folinic acid enhances wound healing in rat model. Adv. Med. Sci..

[34] Kumar S., Chang Y.C., Lai K.H., Hwang T.-L. (2021). Resveratrol, a Molecule with Anti-Inflammatory and Anti-Cancer Activities: Natural Product to Chemical Synthesis. Curr. Med. Chem..

[35] Jia Y., Shi J., Ding B., Zhao L., Xu K., Hu C., Xu W., Zhu A., Yang H., Wang X. (2023). Photoactive Poly-L-Lysine gel with resveratrol-magnesium metal polyphenol network: A promising strategy for preventing tracheal anastomotic complications following surgery. Mater. Today Bio.

[36] Campos R.I., Wu X., Elgland M., Konradsson P., Hammarstrom P. (2016). Novel trans-Stilbene-based Fluorophores as Probes for Spectral Discrimination of Native and Protofibrillar Transthyretin. ACS Chem. Neurosci..

[37] Kim S., Ko H., Park J.E., Jung S., Lee S.K., Chun Y.J. (2002). Design, Synthesis, and Discovery of Novel trans-Stilbene Analogues as Potent and Selective Human Cytochrome P450 1B1 Inhibitors. J. Med. Chem..

[38] Mironov A.N. (2012). Guidelines for Conducting Preclinical Studies of Drugs.

[39] Otranto M., Souza-Netto I., Aguila M.B., Monte-Alto-Costa A. (2009). Male and female rats with severe protein restriction present delayed wound healing. Appl. Physiol. Nutr. Metab..

